# Comparison of the EQ-5D-Y-5L, EQ-5D-Y-3L and PedsQL in children and adolescents

**DOI:** 10.1186/s41687-022-00480-9

**Published:** 2022-06-16

**Authors:** Janine Verstraete, Des Scott

**Affiliations:** 1 Division of Pulmonology, Department of Paediatrics and Child Health, University of Cape Town, Klipfontein Road, Rondebosch, Cape Town, 7700 South Africa; 2 Division of Physiotherapy, Faculty of Health and Rehabilitation Sciences, University of Cape Town, Anzio Road, Observatory, Cape Town, 7925 South Africa

**Keywords:** EQ-5D-Y-3L, EQ-5D-Y-5L, PedsQL, Youth, Concurrent validity

## Abstract

**Background:**

There is an increased use of Patient-Reported Outcome Measures (PROMs) in children and adolescents. The aim of this study was to compare the feasibility, concurrent validity and known-group validity of the EQ-5D-Y-3L (Y-3L), EQ-5D-Y-5L (Y-5L) and PedsQL self-report PROMs.

**Methods:**

Five hundred and fifty children and adolescents, aged 8–15-years, with acute and chronic health conditions and a general population sample were recruited from schools and hospitals in Cape Town South Africa. All respondents self-completed the Y-5L, PedsQL, Self-Rated Health Question and Y-3L. Feasibility of the measures was determined by comparing the number of missing responses. Convergent validity was assessed by Spearman’s and Intra-class correlations on the corresponding items and summary scores respectively. Known-groups validity across health conditions was assessed across the summary scores of the measures with analysis of variance (ANOVA).

**Results:**

The Y-3L and Y-5L had a total of 1% and 3.5% missing responses compared to 19% on the PedsQL. Similar items on the PedsQL and Y-3L/Y-5L showed high correlations (> 0.5) and related items showed moderate correlations (0.3). PedsQL total score was moderately and significantly associated with Y-3L and Y-5L level sum and VAS scores. The Y-3L and Y-5L level sum and VAS scores showed significant differences between known health groups whereas the PedsQL only showed differences between those with acute and chronic illness.

**Conclusion:**

The results of this study show that the Y-3L and Y-5L showed comparable psychometric validity to the PedsQL. When considering the choice between the PedsQL, Y-5L and Y-3L these study results indicate that the EQ-5D-Y instruments (Y-3L and Y-5L) are recommended for studies assessing known-group validity or where missing data should be minimised. The PedsQL generic measure may be preferable in future studies including the general population where a ceiling effect is anticipated. When considering the choice between the Y-5L and the Y-3L there was no systematic difference in the validity between these instruments or between the Y-3L or Y-5L and the PedsQL. Thus, the selection of EQ-5D-Y measures to include in future studies should be guided by the characteristics of the population to be tested.

## Background

Paediatric Patient-Reported Outcome Measures (PROMs) have become increasingly important in health outcomes research with an increase in use in clinical trials and evaluating health systems [1, 2]. Multi-attribute PROMs aim to capture the subjective constructs of health across physical, social and psychological functioning [3, 4]. There are broad categories of measures: disease-specific measures and generic. Disease-specific measures are typically developed to measure the effects of a specific disease or condition [5] and argued to be more responsive in that they detect disease-specific clinical changes [6]. Generic measures can be used in a wide variety of health conditions and the dimensions or items included apply to diverse conditions and populations [4, 6–8]. Thus, generic measures are able to compare health across different health conditions or populations. Generic measures thus have a wider application and can be used in population health surveys, burden of disease studies, epidemiological studies, screening, describing health status, developing management plans for individual patients, informing clinical policy and resource allocation decisions [6, 9–14].

There are currently over 35 published generic PROMs for children and adolescents younger than 18 years [1] of which the EQ-5D-Y and Pediatric Quality of Life Inventory (PedsQL) 4.0 Generic Core scale have been frequently cited [1, 2, 15]. The EQ-5D-Y was adapted from the EQ-5D, an adult measure, to include youth friendly wording and examples [16]. Respondents, aged 8–15 years, can self-report their health, as experienced on that day, across five dimensions and a Visual Analogue Scale (VAS) measuring general health from 0 (worst health) to 100 (best health). The dimensions include mobility, self-care, usual activities, pain or discomfort and emotional state. The original three-level version, EQ-5D-Y-3L (Y-3L), records scores on three levels of severity: no problems, some problems or a lot of problems [16]. The levels of report have recently been expanded to five on the EQ-5D-Y-5L (Y-5L): no/not, a little bit, some/quiet, a lot/really or cannot/extreme(ly) [17]. The increase in levels from three to five levels has been shown to improve the discriminatory power and reduce the ceiling effect of the measure [18, 19].

The PedsQL aims to measure the core dimensions of health, as described by the World Health Organisation (WHO), physical functioning, emotional functioning, social functioning and an additional item of school functioning [20]. The PedsQL has multiple age versions available with questions relevant to the development of the child. The measures for children (aged 8–12 years) and adolescents (aged 13–18 years) both include 23 self-reported items which assess the frequency of problems (never, almost never, sometimes, often, almost always) in the past one month. Although there is overlap between the Y-3L and Y-5L dimensions and the PedsQL items the most notable difference is the inclusion of the school functioning questions on the PedsQL descriptive system. Although ‘going to school’ is included as an example on the Y-3L and Y-5L descriptive system there are no specific questions on academic performance. The expanded Y-5L, with increased level of report, is now more similar to the PedsQL version on this attribute and may show improved association with the PedsQL compared to the Y-3L. The aim of this study was to compare the feasibility, convergent validity, concurrent validity and known group validity of the Y-5L, Y-3L, PedsQL and Self-Rated Health (SRH) question.

## Methods

### Study design and participants

An observational, analytical cohort study was conducted with the Y-5L, Y-3L, PedsQL and SRH question. A head-to-head comparison of the Y-5L and Y-3L instrument performance is presented elsewhere [18, 21].

Three research settings, each with children/adolescents in different health states, were used in Cape Town, South Africa. Although details of socio-economic status were not captured children living in the same geographical area were recruited ensuring that they were from similar socio-economic backgrounds (low to middle income).

Children/adolescents attending two mainstream schools, which admit generally healthy learners without special education needs, were used to recruit a general population sample. Children/adolescents with stable chronic health conditions were recruited from five schools for learners with special education needs. These schools have specialised education services for learners with normal intellect diagnosed with physical disability and/or learning disability. Children/adolescents requiring acute medical treatment were recruited from the inpatient wards of an acute tertiary paediatric hospital and a paediatric orthopaedic hospital.

All children/adolescents aged 8–15 years, who were able to read and write English, the most commonly spoken and written language in South Africa [22], at each facility were eligible for the study. Only those who returned a signed informed consent and assent were included in the study and those who were critically ill or who were medically unstable were excluded as the research may have been too distressing. The sample size was adequately powered (95%) to detect a difference in correlation of scores between the three condition groups with a small effect size 0.4 and a significance of 0.05.

### Instruments

#### EQ-5D-Y

The official Y-3L English version for South Africa was used in this study. The experimental Y-5L English version for the United Kingdom was tested for equivalence in English for South Africa by the EuroQol group [23]. Each version consists of five dimensions namely *Mobility* (walking about), *Looking After Myself* (washing and dressing), *Usual Activities* (going to school, hobbies, sports, playing, doing things with family or friends), *Pain or Discomfort* and *Worried, Sad, or Unhappy*. There is also a general rating of health on a VAS of 0 (worst health) to 100 (best health). The original youth version, Y-3L, describes health on three levels (no problems, some problems and a lot of problems) resulting in 243 (3^5^) health states [16, 24]. The newly expanded version, Y-5L, describes health on five levels [no/not, a little bit, some/quiet, a lot/really, cannot/extreme(ly)] resulting in 3125 (5^5^) health states.

The three or five levels of the descriptive system are expressed with a five-digit code. For example, the Y-3L health state 11223 describes someone with no problems with *Mobility*, no problems with *Looking After Myself*, some problems with *Usual Activities*, some *Pain or Discomfort* and very *Worried, Sad or Unhappy*. The best health state described by the instrument is coded as 11111, describing ‘no problems’ in each of the dimensions [23]. Although the Y-3L has a preference-based score the Y-5L does not [25–27]. As such a level sum score (LSS) was used to describe the responses on the descriptive system where the level labels are treated as numeric data with the best possible score (1 + 1 + 1 + 1 + 1) = 5 and the most severe score for the EQ-5D-Y-3L is (3 + 3 + 3 + 3 + 3) = 15. The other health states will have a LSS ranging between 5 and 15, with a larger score indicating a worse health state. Y-5L is similarly scored with a LSS ranging between 5 and 25 [28]. The LSS is a crude score which does not account for preference of dimensions or weighting of responses [29, 30] but gives some indication of the performance of the dimensions between the Y-3L and Y-5L. Results from Y-3L value sets show that there is a difference in rank order of dimensions and scores attributed to dimensions when compared to the adult EQ-5D-3L [25–27] as such comparing LSS may give a better indication of performance of the Y-3L compared to the Y-5L than using the adult EQ-5D-3L and EQ-5D-5L value sets. The Y-5L VAS was reported for this study.

### Pediatric Quality of Life Inventory (PedsQL)

The 23 item PedsQL 4.0 Generic Core Scales for children aged 8–12 years and 13–18 years were used as appropriate [31]. Both age versions of the PedsQL consist of four dimensions of functioning: physical, emotional, social, and school with 8,5,5 and 5 items respectively. Each item is scored on a Likert scale from 0 to 4 (never a problem, almost never, sometimes, often, or almost always a problem). Items are reversed scored and transformed to a 0–100 scale: 0 = 100, 1 = 75, 2 = 50, 3 = 25, 4 = 0. Dimension scores are calculated by a sum of the item scores divided by the total number of items. A total score is similarly generated by summing the dimension scores over the total number of dimensions giving an overall Health Related Quality of Life (HRQoL) score. Scores for scales with more than 50% missing data are not computed. A higher PedsQL score indicates a better HRQoL [32–34].

### Self-Rated Health (SRH)

The Self-Rated Health (SRH) question asks the child to describe their general health today as: ‘excellent’, ‘very good’, ‘good’, ‘fair’ or ‘poor’. This question has been shown to be a valid measure of subjective health in children and adolescents [35]. The items were scored numerically for data analysis with excellent scored 5 and poor scored 1. The SRH question is expected to capture general health similarly to the EQ-5D-Y VAS [36, 37].

### Procedure

Ethics approval was obtained from the University of Cape Town, Faculty of Health Sciences, Human Research Ethics Committee (HREC 154_2019). The study was carried out in accordance with the declaration of Helsinki involving human participants [38] and the recommended Covid precautions.

Children/adolescents aged 8–15 years admitted to either of the acute inpatient hospital settings were recruited during an onsite visit. For those who were willing and provided consent and assent the parent was asked to complete the socio-demographic information for the child and the children/adolescents were asked to self-complete the Y-5L, PedsQL, SRH and Y-3L in that order. The Y-5L was presented first based on the adult study comparing the EQ-5D-5L and EQ-5D-3L version as it was found that if the EQ-5D-3L was presented first the additional levels on the EQ-5D-5L were not considered [39]. Children and adolescents recruited at one of the hospitals completed the questionnaires in a quiet, private space with supervision from the researcher.

Due to the constraints of the Covid pandemic children and adolescents attending either the mainstream schools or schools for learners with special education needs were recruited through information leaflets that were sent home to them and their parents. For those who were willing and provided consent and assent the instruments were self-completed by the child/adolescent at home under the supervision of their parent. The accompanying information clearly stated that parents should not assist or influence with the completion of the instruments. A reminder was sent out to learners and parents who had not responded after one and two weeks.

### Data management and analysis

#### General performance and feasibility

The Y-5L, Y-3L, PedsQL and SRH responses and descriptive data were summarised in terms of frequency of responses. The feasibility was assessed by comparing the number of missing values across measures.

#### Concurrent validity

The concurrent validity of the dimension scores of the Y-3L and Y-5L were compared to the individual PedsQL items and sub-scale scores using Spearman correlations (r_s_).

It was anticipated that Y-5L/Y-3L *Mobility* dimension would be associated with PedsQL items of hard to walk; 100 m, hard to run and Physical Health Summary Score. Y-5L/Y-3L *Looking After Myself* dimension would be associated with PedsQL hard to bath/shower. Y-5L/Y-3L *Usual Activities* would be associated with participate in sport/exercise, household chores, miss school because not feeling well, miss school to go to the doctor and Y-3L/Y-5L *Worried, Sad or Unhappy* would be associated with items of Sad and Worry. PedsQL summary and total scores were compared to EQ-5D-Y LSS and VAS and scores and SRH scores with the Pearson’s correlation co-efficient. Correlation coefficients were interpreted according to Cohen: 0.1–0.29 low association, 0.3–0.49 moderate association and ≥ 0.5 high association [40].

#### Known-group validity

Children with health conditions receiving acute or chronic health care and those from the general population were compared for known-group validity. Analysis of variance (ANOVA) with Tukey post hoc analysis was used to compare the Y-5L and Y-3LL LSS and VAS scores, PedsQL sub-scales, summary and total scores and the SRH score (which was treated as a scale variable for this analysis).

All data analyses were conducted using SPSS Windows 27.0 (IBM SPSS Inc., Chicago, IL, USA) and Statistica Windows Version 13.0 (TIBCO Software Inc., Palo Alto, CA, USA).

## Results

**Fig. 1:**
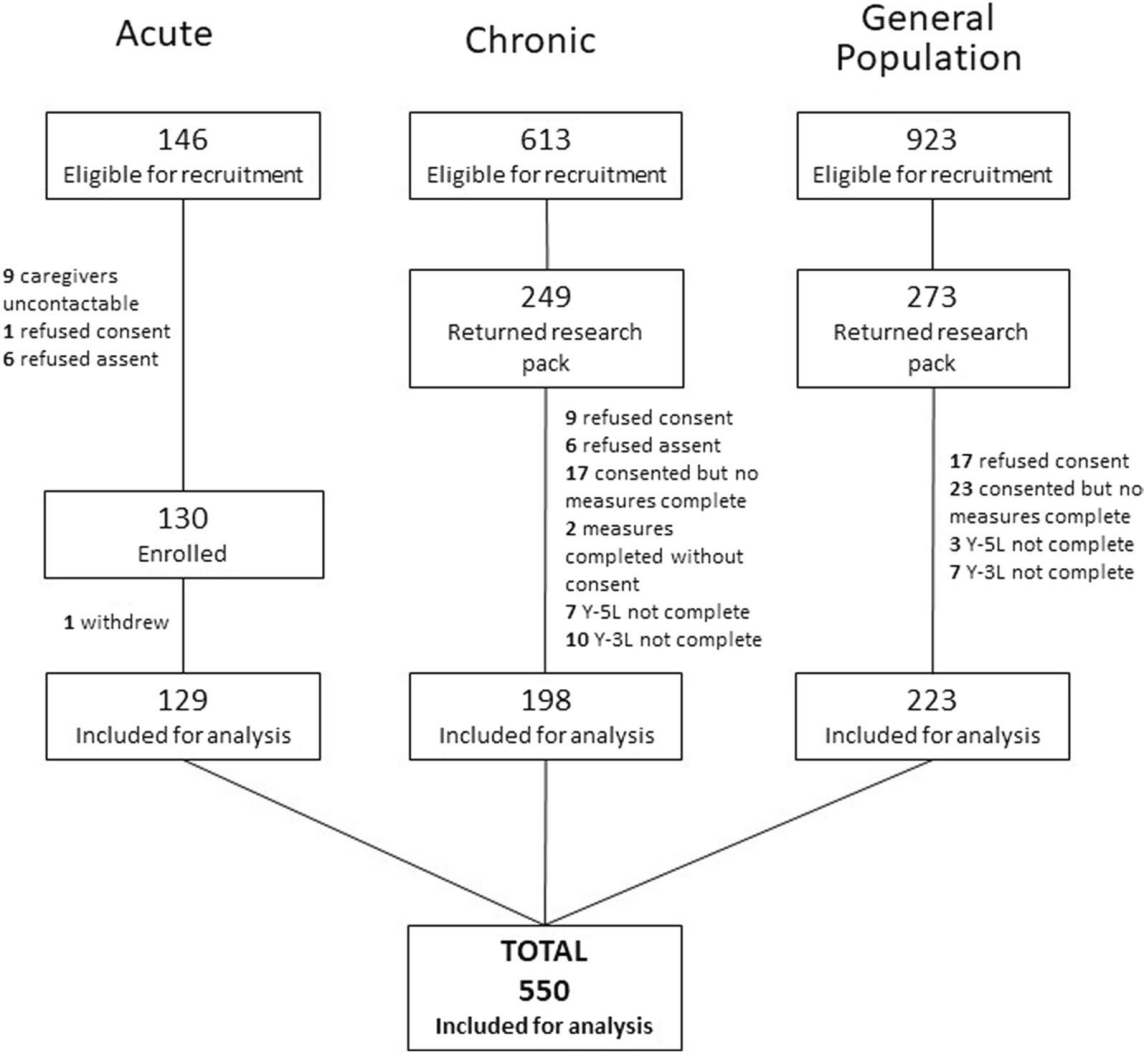
Recruitment into the study

Figure 1 summarises the recruitment and enrolment of participants. The data of 550 children/adolescents has been included for analysis. Reasons for refusal of consent/assent was not collected.

The mean (SD) age of the children/adolescents across the age groups was 11.3 (1.6) years range 8–15 years. The children/adolescents with chronic conditions were older [mean age 12.2 years (SD 1.9 years)] than those in the acute medical setting or from the general population [mean age 11.3 years (SD 1.6 years)] (F = 13.08; p < 0.001). Sex of participants was equally distributed for the overall sample however those with chronic conditions had a higher proportion of males (62%) compared to the general population (40%) (*X*^*2*^ = 20.30; p < 0.001). Most of the children/adolescents needing acute medical management were receiving orthopaedic management whereas those with chronic conditions had a physical disability or learning difficulty (Table 1). The children/adolescents in the general population group had minor health conditions including asthma, eczema and allergy and other conditions including epilepsy, diabetes, glaucoma, and a cardiac lesion.

**Table 1:** Descriptive statistics of the sample

	Acute		Chronic		General population		Total	
	n = 129		n = 198		n = 223		n = 550	
	n	%	n	%	n	%	n	%
*Sex*								
Male	61	47%	123	62%	90	40%	274	50%
*Age*								
years mean (SD)	11.6 (1.9)		12.2 (1.9)		11.3 (1.6)		11.3 (1.6)	
*Medical condition*								
None	0	0%	0	0%	199	89%	199	36%
Physical disability	0	0%	87	44%	0	0%	87	16%
Orthopaedic	83	64%	0	0%	0	0%	83	15%
Learning difficulty*	0	0%	95	48%	0	0%	95	17%
Medical	18	14%	6	3%	1	0%	25	5%
Respiratory, eczema and/allergy	3	2%	7	4%	20	9%	30	5%
Surgical	13	10%	0	0%	0	0%	13	2%
Other^†^	12	9%	3	0	3	1%	18	3%

*Learning difficulties includes attention deficit (hyperactive) disorder, autism spectrum disorder, dyslexia and specific learning disability. ^†^Other includes epilepsy, diabetes, and congenital cardiac lesion

### General instrument performance and feasibility

Table 2 summarises the frequency of responses across the four measures.

**Table 2:** Frequency of responses for the EQ-5D-Y-5L, EQ-5D-Y-3L, Self-Rated Health (SRH) score and PedsQL

Instrument Items	Response options					
	n (%)	n (%)	n (%)	n (%)	n (%)	n (%)
EQ-5D-Y-5L	No	A little bit	Some/quiet	A lot/really	Cannot/extreme	Missing
Mob	360 (65)	85 (15)	24 (4)	5 (1)	74 (13)	2 (0)
LAM	428 (78)	65 (12)	19 (3)	8 (1)	28 (5)	2 (0)
UA	352 (64)	78 (14)	34 (6)	23 (4)	59 (11)	4 (0)
P/D	343 (62)	126 (23)	56 (10)	17 (3)	5 (1)	3 (1)
WSU	348 (63)	136 (25)	31 (6)	12 (2)	13 (2)	10 (2)
VAS mean (SD)	82.2 (22.0)					8 (1)
LSS mean (SD)	8.1 (3.9)					
EQ-5D-Y-3L	No		Some		A lot	Missing
Mob	389 (71)		82 (15)		76 (14)	3 (1)
LAM	435 (79)		85 (15)		29 (5)	1 (0)
UA	370 (67)		108 (20)		70 (13)	2 (0)
P/D	364 (66)		167 (30)		19 (3)	0 (0)
WSU	366 (67)		161 (29)		23 (4)	0 (0)
LSS mean (SD)	6.9 (2.2)					
Self-Rated Health	Excellent	Very good	Good	Fair	Poor	Missing
SRH score	192 (35)	180 (33)	123 (22)	38 (7)	7 (1)	10 (2)
PedsQL	Never	Almost never	Sometimes	Often	Almost always	Missing
Hard to walk; 100 m	311 (57)	54 (10)	68 (12)	19 (3)	75 (14)	23 (4)
Hard to run	257 (47)	57 (10)	67 (12)	34 (6)	111 (20)	24 (4)
Hard to participate in sport/exercise	256 (47)	62 (11)	93 (17)	35 (6)	82 (15)	22 (4)
Hard to lift something heavy	193 (35)	81 (15)	132 (24)	46 (8)	78 (14)	20 (4)
Hard to bath/shower myself	411 (75)	30 (5)	38 (7)	19 (3)	30 (5)	22 (4)
Hard to do household chores	309 (56)	63 (11)	67 (12)	32 (6)	56 (10)	23 (4)
Pain or aches	220 (40)	96 (17)	166 (30)	29 (5)	20 (4)	19 (3)
Low energy levels	259 (47)	87 (16)	113 (21)	37 (7)	32 (6)	22 (4)
Physical Health Summary Score	73.2 (47.8)					
Afraid or scared	246 (45)	93 (17)	143 (26)	23 (4)	25 (5)	20 (4)
Sad	258 (47)	89 (16)	144 (26)	29 (5)	10 (2)	20 (4)
Angry	234 (43)	94 (17)	145 (26)	36 (7)	19 (3)	22 (4)
Trouble sleeping	256 (47)	78 (14)	115 (21)	35 (6)	47 (9)	19 (3)
Worry about what will happen to me	214 (39)	78 (14)	143 (26)	36 (7)	58 (11)	21 (4)
Emotional sub-score mean (SD)	72.0 (22.0)					
Trouble getting along with others	329 (60)	86 (16)	91 (17)	19 (3)	9 (2)	16 (3)
Others don’t what to be my friend	324 (59)	86 (16)	78 (14)	23 (4)	21 (4)	18 (3)
Others tease me	319 (58)	66 (12)	98 (18)	33 (6)	16 (3)	18 (3)
Cannot do things others my age can	248 (45)	74 (13)	114 (21)	42 (8)	51 (9)	21 (4)
Hard to keep up with others	298 (54)	52 (9)	103 (19)	35 (6)	44 (8)	18 (3)
Social sub-score mean (SD)	80 (49)					
Hard to pay attention in class	274 (50)	77 (14)	100 (18)	39 (7)	38 (7)	22 (4)
Forget things	161(29)	102 (19)	171 (31)	51 (9)	45 (8)	20 (4)
Trouble keeping up with schoolwork	262 (48)	75 (14)	111 (20)	39 (7)	42 (8)	21 (4)
Miss school because of not feeling well	247 (45)	98 (18)	119 (22)	29 (5)	37 (7)	20 (4)
Miss school to go doctor or hospital	242(44)	86 (16)	137 (25)	32 (6)	34 (6)	19 (3)
School sub-score mean (SD)	71.0 (23.0)					
Psychosocial summary score mean (SD)	74.0 (23.2)					
Total score mean (SD)	73.8 (236)					

N = 550, *Mob* mobility, *LAM* looking after myself, *UA* Usual Activities; *P/D* pain or discomfort, *WSU* worried, sad or unhappy, *VAS* Visual Analogue Scale, *LSS* Level Sum Score

The Y-5L had a low number of missing scores (0–2%) for individual dimensions with a total of 3.5% of missing responses across all five dimensions and 5% missing for dimension and VAS responses. The Y-3L had an even lower number of missing scores (0–1%) for individual dimensions and only 1% across all dimensions. There were only three children/adolescents with missing responses on the Y-3L dimensions compared to 21 on the Y-5L. Five percent of the participants did not complete the second VAS for the Y-3L. Ten respondents did not complete the SRH question resulting in 2% missingness.

A total of 43 children/adolescents had missing responses on the PedsQL with 16 of them not completing any items. The 16 children who did not complete the measure contributed to a large percentage of missingness thus the missingness in those who did complete the measure (n = 534) resulted in a total of 19% missing responses across the 23 items. The 16 children who did not complete the measure were all from the chronically ill or general population sample and completed the questionnaire at home, there were no other relevant demographic factors for this group. The number of missing responses at an item level for those who did complete the questionnaire ranged from 0 to 1%. The missing items were highest for the 8 items of physical health with 9% missingness compared to 4%, 2% and 4% for the 5 items in the emotional, social, and school sub-scales respectively.

### Concurrent validity

Table 3 shows that the Y-3L and Y-5L had similar high association with similar items on the PedsQL generic measure and moderate associations with related items. Table 4 shows the concurrent validity of the Y-3L and Y-5L VAS and LSS with the PedsQL and SRH scores. All Y-3L and Y-5L scores were moderately associated with the SRH score across condition groups. The PedsQL total score was moderately and significantly associated with the Y-3L/Y-5L LSS and VAS scores across all condition groups, except for the VAS score in the group of children with acute illness. The Y-3L and Y-5L LSS, physical and psychosocial summary scores had significant moderate associations for children/adolescents with a stable chronic condition and for the general population. The emotional and social sub-scale scores showed greater association with the Y-5L and Y-3L scores than the school sub-scale. The Y-3L and Y-5L VAS and LSS showed greater association with the SRH than the PedsQL for children with a chronic condition and from the general population. The PedsQL had a weak and non-significant correlation with the SRH score for children with an acute condition whereas the Y-3L and Y-5L both showed moderate significant correlations.

**Table 3:** Spearman correlation of EQ-5D-Y-5L and EQ-5D-Y-3L dimension scores and PedsQL item and sub-scale scores

PedsQL	EQ-5D-Y-5L					EQ-5D-Y-3L				
	Mob	LAM	UA	P/D	WSU	Mob	LAM	UA	P/D	WSU
Hard to walk; 100 m	− 0.61**	− 0.40**	− 0.54**	− 0.32**	− 0.21**	− 0.61**	− 0.35**	− 0.60**	− 0.26**	− 0.20**
Hard to run	− 0.60**	− 0.35**	− 0.67**	− 0.31**	− 0.21**	− 0.63**	− 0.34**	− 0.67**	− 0.25**	− 0.18**
Hard to participate in sport/exercise	− 0.54**	− 0.34**	− 0.63**	− 0.30**	− 0.20**	− 0.55**	− 0.34**	− 0.65**	− 0.21**	− 0.16**
Hard to lift something heavy	− 0.33**	− 0.34**	− 0.33**	− 0.21**	− 0.10*	− 0.33**	− 0.31**	− 0.32**	− 0.17**	− 0.11*
Hard to bath/shower myself	− 0.35**	− 0.60**	− 0.32**	− 0.21**	− 0.14**	− 0.43**	− 0.52**	− 0.41**	− 0.18**	− 0.16**
Hard to do household chores	− 0.36**	− 0.40**	− 0.35**	− 0.23**	− 0.11*	− 0.35**	− 0.42**	− 0.39**	− 0.20**	− 0.08
Pain or aches	− 0.24**	− 0.23**	− 0.25**	− 0.54**	− 0.24**	− 0.26**	− 0.23**	− 0.29**	− 0.50**	− 0.27**
Low energy levels	− 0.30**	− 0.24**	− 0.36**	− 0.32**	− 0.27**	− 0.32**	− 0.25**	− 0.42**	− 0.31**	− 0.21**
Physical Health Summary Score	− 0.60**	− 0.47**	− 0.62**	− 0.40**	− 0.26**	− 0.61**	− 0.44**	− 0.66**	− 0.36**	− 0.23**
Afraid or scared	− 0.13**	− 0.12**	− 0.10*	− 0.20**	− 0.30**	− 0.12**	− 0.14**	− 0.14**	− 0.17**	− 0.33**
Sad	− 0.04	− 0.02	− 0.05	− 0.05**	− 0.37**	− 0.05	− 0.03	− 0.09*	− 0.18**	− 0.33**
Angry	− 0.01	− 0.05	0.01	− 0.15**	− 0.26**	0.00	− 0.09*	0.00	− 0.20**	− 0.32**
Trouble sleeping	− 0.01	− 0.05	− 0.09*	− 0.22**	− 0.24**	− 0.04	− 0.08	− 0.10*	− 0.20**	− 0.23**
Worry about what will happen to me	− 0.12**	− 0.05	− 0.16**	− 0.23**	− 0.29**	− 0.10*	− 0.06	− 0.20**	− 0.25**	− 0.34**
Emotional sub-score	− 0.11**	− 0.09*	− 0.14**	− 0.28**	− 0.42**	− 0.11*	− 0.13**	− 0.18**	− 0.29**	− 0.45**
Trouble getting along with other kids/teenagers	− 0.15**	− 0.04	− 0.15**	− 0.13**	− 0.16**	− 0.09*	− 0.10*	− 0.13**	− 0.11*	− 0.15**
Other kids/teenagers do not what to be my friend	− 0.15**	− 0.14**	− 0.20**	− 0.18**	− 0.22**	− 0.09*	− 0.19**	− 0.17**	− 0.17**	− 0.22**
Other kids/teenagers tease me	− 0.18**	− 0.18**	− 0.16**	− 0.18**	− 0.21**	− 0.14**	− 0.21**	− 0.16**	− 0.21**	− 0.21**
Cannot do things others my age can do	− 0.42**	− 0.36**	− 0.45**	− 0.23**	− 0.20**	− 0.43**	− 0.34**	− 0.49**	− 0.21**	− 0.17**
Hard to keep up with others	− 0.48**	− 0.33**	− 0.51**	− 0.20**	− 0.14**	− 0.44**	− 0.36**	− 0.51**	− 0.17**	− 0.10*
Social sub-score	− 0.42**	− 0.33**	− 0.46**	− 0.26**	− 0.27**	− 0.39**	− 0.36**	− 0.46**	− 0.25**	− 0.24**
Hard to pay attention in class	0.04	− 0.06	− 0.05	− 0.10*	− 0.22**	0.05	− 0.08	− 0.09*	− 0.11**	− 0.19**
Forget things	− 0.01	− 0.07	− 0.07	− 0.10*	− 0.16**	− 0.05	− 0.13**	− 0.12**	− 0.09*	− 0.14**
Trouble keeping up with my schoolwork	− 0.05	− 0.15**	− 0.16**	− 0.14**	− 0.20**	− 0.09*	− 0.14**	− 0.20**	− 0.11*	− 0.17**
Miss school because of not feeling well	− 0.29**	− 0.21**	− 0.35**	− 0.32**	− 0.17**	− 0.33**	− 0.24**	− 0.36**	− 0.25**	− 0.15**
Miss school to go doctor or hospital	− 0.44**	− 0.29**	− 0.43**	− 0.31**	− 0.15**	− 0.44**	− 0.27**	− 0.43**	− 0.21**	− 0.11*
School sub-score	− 0.23**	− 0.22**	− 0.32**	− 0.26**	− 0.27**	− 0.26**	− 0.26**	− 0.36**	− 0.21**	− 0.23**

n = 533, *p < 0.05 and **p < 0.001 (2-tailed). Cells shaded in grey have a moderate association > 0.30, correlations are negative as a higher PedsQL score indicates a better HRQoL. 1 PedsQL score not computed as > 50% missing data and 16 respondents did not complete the PedsQL *Mob* mobility; *LAM* looking after myself, *UA* usual activities, *P/D* pain or discomfort, *WSU* worried, sad or unhappy, *VAS* Visual Analogue Scale

**Table 4:** Summary of concurrent validity with the PedsQL summary and sub-scores, Self-Rated Health and EQ-5D-Y-3L and EQ = 5D-Y-5L LSS and VAS scores with Pearson correlation

			PedsQL						Self-Rated Health
			Total	Summary scores					
				Physical	Psychosocial	Psychosocial sub-scores			
						Emotional	Social	School	
Acute (n = 129)	EQ-5D-Y-5L	VAS	0.08	0.14	0.08	− 0.04	0.02	0.03	0.41**
		LSS	− 0.34**	− 0.51**	− 0.14	− 0.29**	− 0.01	− 0.19*	− 0.33**
	EQ-5D-Y-3L	LSS	− 0.34**	− 0.46**	− 0.21*	− 0.26**	− 0.03	− 0.21*	− 0.33**
	SRH		0.17	0.13	0.16	0.17	0.22**	0.40	
Chronic (n = 198)	EQ-5D-Y-5L	VAS	0.35**	0.29**	0.36**	0.12	0.25**	0.31**	0.44**
		LSS	− 0.54**	− 0.69**	− 0.23**	− 0.39**	− 0.23**	− 0.36**	− 0.33**
	EQ-5D-Y-3L	LSS	− 0.58**	− 0.73**	− 0.29**	− 0.39**	− 0.25**	− 0.39**	− 0.43**
	SRH		0.36**	0.38**	0.28**	0.34**	0.16**	0.16**	
GP (n = 223)	EQ-5D-Y-5L	VAS	0.37**	0.34**	0.35**	0.21**	0.26**	0.34**	0.67**
		LSS	− 0.44**	− 0.38**	− 0.49**	− 0.30**	− 0.21**	− 0.42**	− 0.42**
	EQ-5D-Y-3L	LSS	− 0.52**	− 0.43**	− 0.54**	− 0.32**	− 0.33**	− 0.50**	− 0.53**
	SRH		0.34**	0.30**	0.32**	0.33**	0.13	0.31**	
Total (n = 550)	EQ-5D-Y-5L	VAS	0.33**	0.37**	0.23**	0.16**	0.25**	0.27**	0.51**
		LSS	− 0.55**	− 0.69**	− 0.22**	− 0.44**	− 0.32**	− 0.40**	− 0.39**
	EQ-5D-Y-3L	LSS	− 0.58**	− 0.69**	− 0.29**	− 0.44**	− 0.34**	− 0.44**	− 0.45**
	SRH		0.37**	0.35**	0.33**	0.30**	0.24**	0.24**	

^*^p < 0.05 and **p < 0.001 level (2-tailed). Cells shaded in grey have a significant moderate association > 0.30, *VAS* Visual Analogue Scale, *LSS* Level Sum Score, *SRH* Self-Rated Health, *GP* general population

### Known-group validity

Table 5 presents the known-group validity of those with acute or chronic health conditions and the general population. The Y-5L (LSS and VAS), Y-3L (LSS and VAS) and PedsQL Physical Health Summary Score were able to detect significant differences between health groups. The PedsQL Psychosocial Health Summary Score and the PedsQL Total scores were able to detect differences between the general population and ill health, but not between acute and chronic groups.

**Table 5:** Comparison of known-group validity for health condition across the EQ-5D-Y-5L, EQ-5D-Y-3L, PedsQL and Self-Rated Health (SRH) question

Mean (SD)	Health condition				Acute versus GP	Chronic versus GP
	Acute	Chronic	GP	Acute versus chronic		
EQ-5D-Y-5L	N = 129	N = 198	N = 223	p-value	p-value	p-value
LSS	11.6 (4.6)	8.4 (3.5)	8.8 (1.6)	< 0.001	< 0.001	< 0.001
VAS	67.7 (28.2)	84.1 (19.8)	89.1 (14.5)	< 0.001	< 0.001	0.034
EQ-5D-Y-3L	N = 129	N = 198	N = 223			
LSS	8.7 (2.6)	7.1 (2.0)	5.6 (1.0)	< 0.001	< 0.001	< 0.001
PedsQL	N = 128	N = 189	N = 216			
Physical Health Summary Score	53.7 (26.0)	66.7 (24.8)	86.2 (14.3)	< 0.001	< 0.001	< 0.001
Psychosocial Health Summary Score	69.2 (16.0)	65.4 (17.8)	82.8 (13.4)	0.090	< 0.001	< 0.001
Emotional sub-score	74.2 (20.1)	64.8 (23.5)	76.6 (18.9)	< 0.001	0.559	< 0.001
Social sub-score	72.1 (20.5)	68.0 (22.2)	89.4 (14.1)	0.149	< 0.001	< 0.001
School sub-score	61.2 (24.3)	63.3 (22.4)	82.3 (16.0)	0.640	< 0.001	< 0.001
PedsQL Total Score	65.3 (16.4)	65.7 (17.0)	83.6 (12.7)	0.970	< 0.001	< 0.001
	N = 129	N = 193	N = 218			
SRH	3.7 (1.0)	3.8 (1.0)	4.2 (0.9)	0.386	< 0.001	< 0.001

*GP* general population, *LSS* level sum score, *VAS* Visual Analogue Scale, *SRH* Self-Rated Health

## Discussion

The aim of this study was to investigate the feasibility, concurrent validity and known group validity of the Y-3L, Y-5L, PedsQL and SRH.

To our knowledge, this was the first study to compare the Y-3L and Y-5L to the PedsQL generic measure and SRH. Children/adolescents receiving acute medical management and with a stable chronic condition were considered a suitable population for comparison of the measures as there was a spread of disease severity, and consequently a spread of scores. Furthermore, the Y-3L and PedsQL [41–44] and Y-3L and SRH [37, 45] have been compared in previous studies.

The number of missing responses was higher on the longer PedsQL measure (18%) than the shorter measures of Y-5L (3.5%), Y-3L (1%) or SRH (2%), when the respondents who did not complete any PedsQL items were excluded. This is further evident from the number of respondents with missing responses with 27 on the PedsQL, 21 on the Y-5L, 10 on the SRH and 3 on the Y-3L. This highlights that it is potentially the number of items and the number of responses which contributes to the missing data. Reasons for missing data was not recorded and cannot be commented on.

The distribution of responses between Y-3L and Y-5L and PedsQL are difficult to compare. The Y-3L/Y-5L asks the respondents to rate dimensions on a severity scale for today whereas the PedsQL asks about the frequency of problems for the last month. Although there is overlap in items/dimensions the reporting of them is different and as such the distribution will differ across the five/three levels of report. Despite these differences in the descriptive systems there were high correlations between Y-3L/Y-5L dimensions and similar items on the PedsQL. There was further moderate correlation with related items e.g. mobility also showed correlations with “hard to bath/shower”, “doing household chores”, “low energy”, “hard to keep up with others” etc. Although it was postulated that the Y-5L would show higher association with the PedsQL than the Y-3L there was no systematic difference between them. Most of the other studies compared Y-3L dimension scores to PedsQL sub-scores or total scores [41, 44, 45] except Scalone et al. [43] who found a low to moderate correlation on similar items on a large sample of general population and small number of ill children. Comparison of Y-3L dimensions and PedsQL sub-scores reported moderate correlation in general population samples [44] and those with health conditions [36, 41]. Both the Y-3L and the PedsQL were used in children with acute Thalassemia with acceptable reliability on Cronbach’s alpha but no comparison of performance between the two measures was made [46]. Similarly, the EQ-5D-Y-5L and PedsQL both showed a decrement in HRQoL with sleep deprivation, physical activity and screen time in a large general population sample in Hong Kong but no comparison between instrument performance was made [47].

Although the EQ-5D-Y descriptive system does not explicitly include school function there were moderate correlations between PedsQL items of “missing school because of not feeling well”, “missing school to go the doctor and hospital”, the school sub-score and the Y-3L and Y-5L dimension of *Usual Activities*. There was low but significant with *Usual Activities* and the PedsQL item of ‘trouble keeping up with schoolwork.” Furthermore the LSS of the Y-3L and Y-5L showed moderate correlations showing that EQ-5D-Y dimensions do in fact capture school functioning.

The PedsQL Total Score had a greater correlation with the Y-3L/Y-5L LSS than the VAS scores which was to be expected as the VAS measures general health and may include other influencers of health which are not captured on the dimensions or items of the corresponding measures. The association between Y-3L/Y-5L LSS was greater for those with chronic conditions or from the general population. Although the Physical Health scores showed moderate to strong correlations for children across health groups the Psychosocial scores showed a weak to moderate correlation, with weaker correlations in those with an acute or chronic condition compared to the general population. This cannot be compared to other studies as they did not look at comparison of instruments across health groups but rather for the entire sample [36, 41, 43, 44]. This could be attributed to the recall period of Today versus that of the past one month. The EQ-5D-Y is more sensitive in capturing acute health problems or those with stable health condition (such as with a stable chronic illness) but may miss those with fluctuating health over time [48]. As such the PedsQL condition specific acute or chronic measures may be more appropriate to use with the disadvantage that you cannot directly compare different health conditions [9]. Despite this the Y-5L and Y-3L LSS were able to differentiate between health conditions (acute, chronic and general population). Whereas the PedsQL showed no difference between those with acute or chronic illness. This was similarly found in the Psychosocial Health Summary Score, however the Physical Health Summary Score showed significant differences between the three groups.

The SRH Question showed stronger correlation to the VAS across all condition groups which was similarly reported in a Swedish study with the Y-3L VAS [37]. The correlation was stronger in the general population group indicating that the VAS may be more sensitive in detecting differences in general health than the five levels of the SRH item. This is confirmed in this study in that VAS was significantly different between those with different health conditions but there was no difference between those with acute and chronic health problems on the SRH question.

Due to the limitations of the Covid pandemic on recruitment of children/adolescents at schools there may be non-response bias. Although parents and children/adolescents were explicitly instructed to complete the measures on their own without influence from others there was no way to ensure this in the sample with a chronic condition and general population. The Correlation of the Y-3L, Y-5L, PedsQL and SRH may have been influenced by the relatively high ceiling effect, most notably for the General population sample. The non-randomised order of the questionnaires could be influencing the results and contributing to an order effect. As the Y-5L is a newly developed measure it has first been tested in English, the source language, in South Africa before translation and adaptation into the other eleven South African languages. Although English is widely spoken and written in South Africa inclusion of only English questionnaires could have excluded parts of the population who speak or write in one of the other ten official languages of South Africa. No details on ethnicity were collected on which to judge the generalisability of the sample however, no one was excluded based on gender, race or religion.

## Conclusion

The results of this study show that the Y-3L and Y-5L showed comparable psychometric validity to the PedsQL. When considering the choice between the PedsQL, Y-5L and Y-3L these study results indicate that theEQ-5D-Y instruments (Y-3L and Y-5L) are recommended for studies assessing known-group validity, particularly between children with and without illness, or where missing data should be minimised. The PedsQL generic measure does not capture acute health problems but shows improved performance specifically with a lower ceiling effect and spread of responses in those with a more stable health condition. As such the PedsQL generic measure may be preferable in future studies including the general population. Distribution of responses across the Y-3L, Y-5L and PedsQL cannot be compared and comparison of the Y-3L to the Y-5L was not the aim of this paper.

When considering the choice between the Y-5L and the Y-3L there was no systematic difference in the validity between these instruments or between the Y-5L or Y-3L and the PedsQL. Thus, the selection of EQ-5D-Y measure for future studies should be guided by the characteristics of the population to be included, for example the Y-5L may be preferred in a population where a higher ceiling effect is anticipated. In contrast the Y-3L may be preferred in a study which includes a younger cohort or where a lower literacy level is anticipated. Further research is recommended comparing the performance of the PedsQL and the Y-3L and Y-5L in homogenous disease groups to guide future researchers on the selection of the most appropriate instrument.

## Data Availability

The datasets used and/or analysed during the current study are available from the corresponding author on reasonable request.

## Abbreviations

ANOVA: Analysis of variance
HRQoL: Health Related Quality of Life
PROMs: Patient-Reported Outcome Measures
PedsQL: Pediatric Quality of Life Inventory
SRH: Self-Rated Health
VAS: Visual Analogue Scale
WHO: World Health Organisation
Y-3L: EQ-5D-Y-3L
Y-5L: EQ-5D-Y-5L
